# Derivation of human primary prostate epithelial cell lines by differentially targeting the *CDKN2A* locus along with expression of hTERT

**DOI:** 10.21203/rs.3.rs-4294058/v1

**Published:** 2024-05-06

**Authors:** Jason S. Wasserman, Holly Fowle, Rumesa Hashmi, Diba Atar, Kishan Patel, Amir Yarmahmoodi, Alexander W. Macfarlane, Yinfei Tan, Edna Cukierman, Bojana Gligorijevic, Adam Karami, Kelly A. Whelan, Kerry S. Campbell, Xavier Graña

**Affiliations:** 1 Fels Cancer Institute for Personalized Medicine; 2 Temple University Lewis Katz School of Medicine, Philadelphia, PA; 3 Institute for Cancer Research, Cancer Signaling & Microenvironment program, Fox Chase Cancer Center; 4 Bioengineering Department, Temple University, Philadelphia PA

## Abstract

Prostate cancer (PCa) is the most common cancer diagnosed in men worldwide and the second leading cause of cancer-related deaths in US males in 2022. Prostate cancer also represents the second highest cancer mortality disparity between non-Hispanic blacks and whites. However, there is a relatively small number of prostate normal and cancer cell lines compared to other cancers. To identify the molecular basis of PCa progression, it is important to have prostate epithelial cell (PrEC) lines as karyotypically normal as possible. Our lab recently developed a novel methodology for the rapid and efficient immortalization of normal human PrEC that combines simultaneous CRISPR-directed inactivation of *CDKN2A* exon 2 (which directs expression of p16^INK4A^ and p14^ARF^) and ectopic expression of an *hTERT* transgene. To optimize this methodology to generate immortalized lines with minimal genetic alterations, we sought to target exon 1α of the *CDKN2A* locus so that p16^INK4A^ expression is ablated while p14^ARF^ expression remains unaltered. Here we describe the establishment of two cell lines: one with the above-mentioned p16^INK4A^ only loss, and a second line targeting both products in the *CDKN2A* locus. We characterize the potential lineage origin of these new cell lines along with our previously obtained clones, revealing distinct gene expression signatures. Based on the analyses of protein markers and RNA expression signatures, these cell lines are most closely related to a subpopulation of basal prostatic cells. Given the simplicity of this one-step methodology and the fact that it uses only the minimal genetic alterations necessary for immortalization, it should also be suitable for the establishment of cell lines from primary prostate tumor samples, an urgent need given the limited number of available prostate cancer cell lines.

## Introduction

Prostate cancer (PCa) is the most common cancer diagnosed in men worldwide, accounting for an estimated 14.1% of new cancer cases and 6.8% of cancer deaths in 2022 ^1,2^. Prostate cancer also represents the second highest cancer mortality disparity rates between non-Hispanic blacks, and whites ^3^. However, the molecular basis of prostate cancer development and the underlying disparities remain unclear. Relative to other cancers, it has been particularly challenging to develop prostate cell line models in culture to help address this gap in knowledge ^4^. As with most other primary human cell types, prostate epithelial cells (PrEC) undergo a limited number of passages before they become senescent, a process in which cells stop dividing and exhibit distinctive phenotypic changes ^5^. Immortalization allows cells to bypass senescence and continue to divide in culture. While ectopic expression of human telomerase reverse transcriptase (hTERT) is effectively used for the immortalization of various normal human cell types, immortalization of primary PrEC with hTERT alone has had very limited success, pointing to further alterations being required for efficient immortalization ^6^. *CDKN2A*/p16^INK4A^ downregulation via mechanisms including promoter methylation, has been suggested, possibly facilitating the immortalization of the few existing examples of hTERT-immortalized hPrEC ^6,7^.

We have recently developed an immortalization strategy that combines the exogenous expression of an *hTERT* transgene with CRISPR-mediated inactivation of the *CDKN2A* exon 2 locus ^8^. This locus directs the expression of both p16^INK4A^ and p14^ARF^ by sharing a common intron translated in alternate reading frames. The α transcript encodes the p16^INK4A^ and the β transcript specifies the alternative product p14^ARF 9,10^. This methodology resulted in the successful establishment of two independently derived immortalized “normal” (i.e. non-transformed) hPrEC clones that based on marker expression (namely cytokeratin 5 and transcription factor p63) are likely to be of a basal cell origin ^8^. These immortal PrEC exhibit fundamental characteristics of normal cells, including diploid genomes, normal p53 and pRB cell responses, the ability to form non-invasive spheroids, and a non-transformed phenotype. Moreover, our methodology was used to successfully immortalize patient-derived prostate cancer cells from a man of Caribbean descent, indicating that the strategy is also suitable for the establishment of prostate cancer cell lines ^11^. Cytogenetic analysis of the two immortalized PrEC revealed a near-normal karyotype that was also homogeneous, given the presumed clonal nature of these cell lines. This is a significant advantage over other hPrEC lines such as EP156T ^6^, which exhibit several abnormalities that could prove confounding in oncogene/tumor suppressor cooperativity, cell transformation, and/or tumorigenicity studies.

However, others have shown that the inactivation of p53, which is downstream of p14^ARF^, with a dominant negative mutant promotes proliferation but is not essential for the immortalization of PrEC expressing hTERT with knockdown p16^INK4 12^. Thus, we sought to optimize our immortalization methodology further by eliminating all non-essential genetic alterations required for immortalization. We reasoned that ectopic hTERT expression combined with p16^INK4A^ knockout alone (via CRISPR-directed targeting of *CDKN2A* exon 1α) would be necessary and sufficient to achieve immortalization. Using a multipronged approach, our results show that the two new clones described here and the two that we generated previously ^8^ likely originated from a basal prostate lineage.

Hence, we herein introduce two novel PrEC human models and the needed methodologies to generate these and other types of cellular models that could serve the scientific community.

## Results

### Derivation of new normal PrEC immortal cell lines

**Figure 1.**
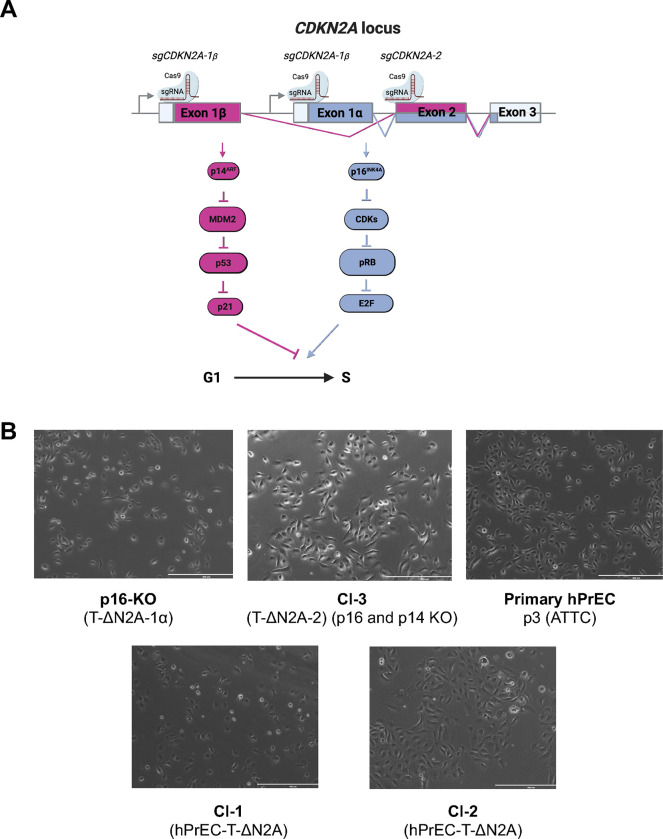
Generation of novel immortal hPrEC cell lines designated p16-KO and Cl-3. **(A)** Scheme of the *CDKN2A* gene locus, which encodes two unrelated proteins p14^ARF^ and p16^INK4A^ via two separate promoters and alternative splicing of Exons 1α and 1β. Note that exon 2 is common but read in alternative frames. p14^ARF^ and p16^INK4A^ trigger two district senescence pathways that converge to induce G1 arrest. Two sgRNAs target exon 1α and 1β, which encode portions of p16^INK4A^ and p14^ARF^ proteins, respectively, another sgRNA targets the shared exon 2. **(B)** Morphology of primary hPrEC in culture and cell lines p16-KO and Cl-3 obtained upon selection with antibiotics. Each cell line was derived from a single colony, but the cell lines have not been single cell cloned, as they do not tolerate growth at low density. Cl-1 and Cl-2 were described previously.

We designed guide RNAs (sgCDKN2A-1α and sgCDKN2A-1β) for Cas9 to selectively target *CDKN2A* exons 1α and 1β to knockout p16^INK4A^ and p14^ARF^, respectively (Fig. 1A). hPrECs at passage 4, were transduced with lentiviruses expressing hTERT and/or sgCDKN2A1α/Cas9 or sgCDKN2A1β/Cas9 and selected with hygromycin and/or puromycin. We also transduced hPrEC with lentiviruses expressing hTERT and sgCDKN2A-2/Cas9 ^8^ and selected colonies using antibiotic treatment to confirm the reproducibility of the original methodology. One colony of the combination of ectopic hTERT expression and *CDKN2A* exon 1α knockout designated T-ΔN2A-1α (p16-KO) and one combining ectopic hTERT expression and *CDKN2A* exon 2 knockout designated T-ΔN2A-2/Clone-3 (Cl-3) were successfully selected. These colonies were not single cell selected, because the cells do not grow well if seeded at very low densities. No colonies transduced with vectors encoding hTERT and inactivation of the *CDKN2A* exon 1β locus survived selection, supporting the notion that p14^ARF^ knockout is insufficient to impede senescence signaling. Consistent with previous observations made in our lab and by others, no colonies transduced with hTERT lentiviruses alone survived selection. This indicates that the introduction of a single hTERT transgene is inefficient as a single modality for immortalization of PrEC; even if these are transduced at passage 4 and grown under optimal conditions, supporting why long-term successful stable immortalization of PrEC has been rarely accomplished ^6,7,13^. Non-transduced primary hPrEC grew sparsely, with cells varying in size at passage 3 (Fig. 1B). Starting from passage 7, the majority of hPrEC showed senescence characteristics with enlarged cell nuclei and flat morphology, and by passage 8, few cells remained attached (not shown). In contrast, the two newly derived p16-KO and Cl-3 cell lines continued to proliferate, relatively fast, and maintained a morphology akin to that noted in the parental PrEC cells (Fig. 1B). Similar cellular characteristics were also noted to be preserved in the previously derived clones Cl-1 and Cl-2 (Fig. 1B). Since their derivation, the two immortalized cell lines have been cultured continuously without showing signs of senescence (>passage 44).

### CRISPR/Cas9 sgRNAs effectively inactivated their target exons within the *CDKN2A* locus.

**Figure 2.**
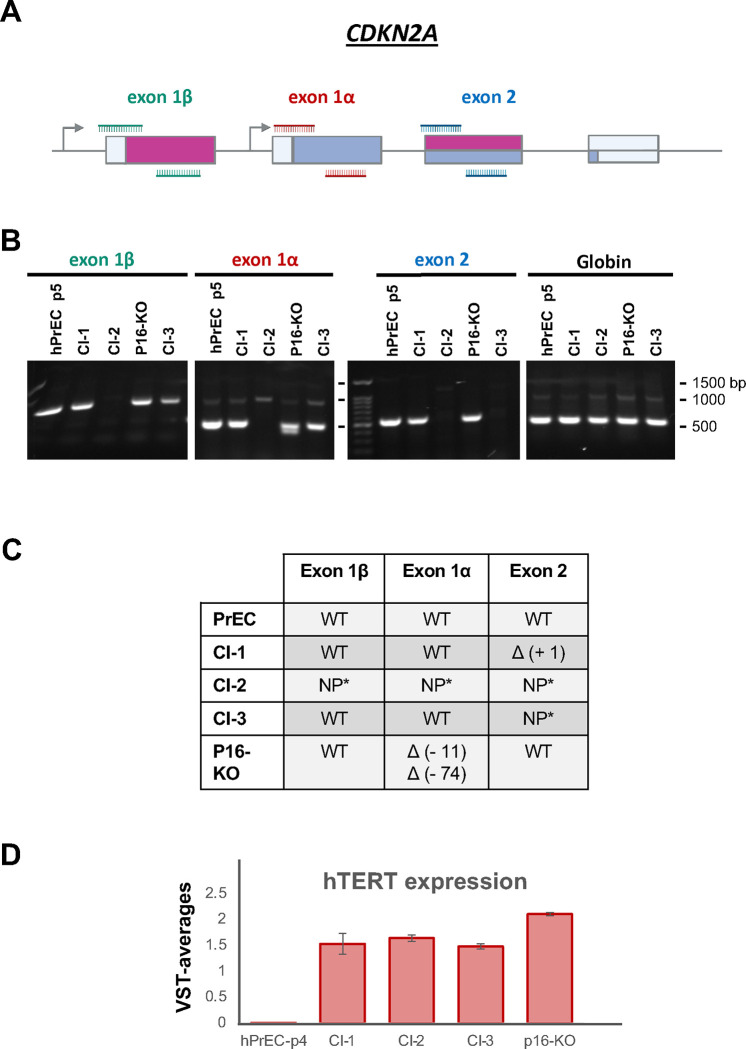
Cas9-CRISPR effectively targeted the *CDKN2A* locus. **A.** Schematic representation of the *CDKN2A* locus and primer sets flanking the sgRNA target sites. **B.** PCR amplification of the *CDKN2A* locus with primer sets flanking predicted Cas9 cut sites. Genomic DNA obtained from each clone and PrEC was amplified with the primer sets indicated above and resolved by agarose electrophoresis. A DNA ladder is shown in the first lane of the Exon 2 gel. All products match their predicted sizes. Gel Images are cropped for clarity. Images corresponding to full-length gels are shown in Suppl. Fig. 1B. **C.** Summary alterations determined by sanger sequencing of the PCR-amplified products (see Suppl. Fig 1). *NP* signifies lack of amplified PCR product. **D.** hTERT is overexpressed in all the cell lines. hTERT mRNA expression was determined by RNA-Seq as described in the text and Fig. 6. Represented is the variance stabilization transformed normalized average (VST-averages) for hTERT in PrEC compared to all the immortalized cell lines.

Genetic alterations triggered by the sgRNAs were characterized by PCR analysis of genomic DNA (gDNA) from clones p16-KO and Cl-3 in comparison to our previous Cl-1 and Cl-2 ^8^ and to the parental hPrEC, using specific primers flanking the CRISPR target cut sites (Fig. 2A). We had previously shown that Cl-1 gDNA amplified with primers flanking exon 2 generates a slightly slower migrating PCR product than the band obtained with gDNA from control PrEC, indicating a very small insertion during repair following Cas9 cleavage ^8^. In contrast, Cl-2 gDNA did not produce any bands after amplification, as the region deleted is much larger than the region that was selected for amplification, consistent with findings using chromosome microarray analysis (CMA) ^8^. Fig. 2B shows that Cl-3 gDNA was amplified with primer sets for both exon 1α and exon 1β, but no PCR products were generated with the primer set for Exon 2, indicating that a deletion spans at least a region complementary to one of the primers. Clone p16-KO produced a double band for exon 1α, indicating differential alteration of the alleles (Fig. 2B). The faster migrating band indicates a small deletion, while the upper band may correspond to a product with a much smaller alteration not detectable by PCR. As expected, Cl-2 did not produce any bands and Cl-1 generated a band with all primers (Fig. 2B). Sanger-sequencing of the amplified products revealed that the p16-KO cell line has allele-specific deletions of 11 and 74 nucleotides (Fig. 2C, Suppl. Fig 1). As Cl-3 did not amplify with primers flanking the exon-2 sgRNA target site and no alterations were found in exons 1α and 1β, an alteration is predicted to affect a region spanning one or both flanking primers. Sequencing of Cl-1 revealed the insertion of a single nucleotide. Lastly, Cl-2 was not amplified with any primer set indicating a large deletion spanning the whole *CDKN2A* locus, which is consistent with the Chromosomal array data that we previously reported for this clone ^8^. Moreover, RNA-Seq data from PrEC and the immortal cell lines demonstrates expression of hTERT in all the clones, but not in PrEC (Fig. 2D) (RNA-Seq approach is described later in the results).

### p16-KO and Cl-3 express basal markers and diminishing expression of the CK18 luminal marker with passage.

**Figure 3.**
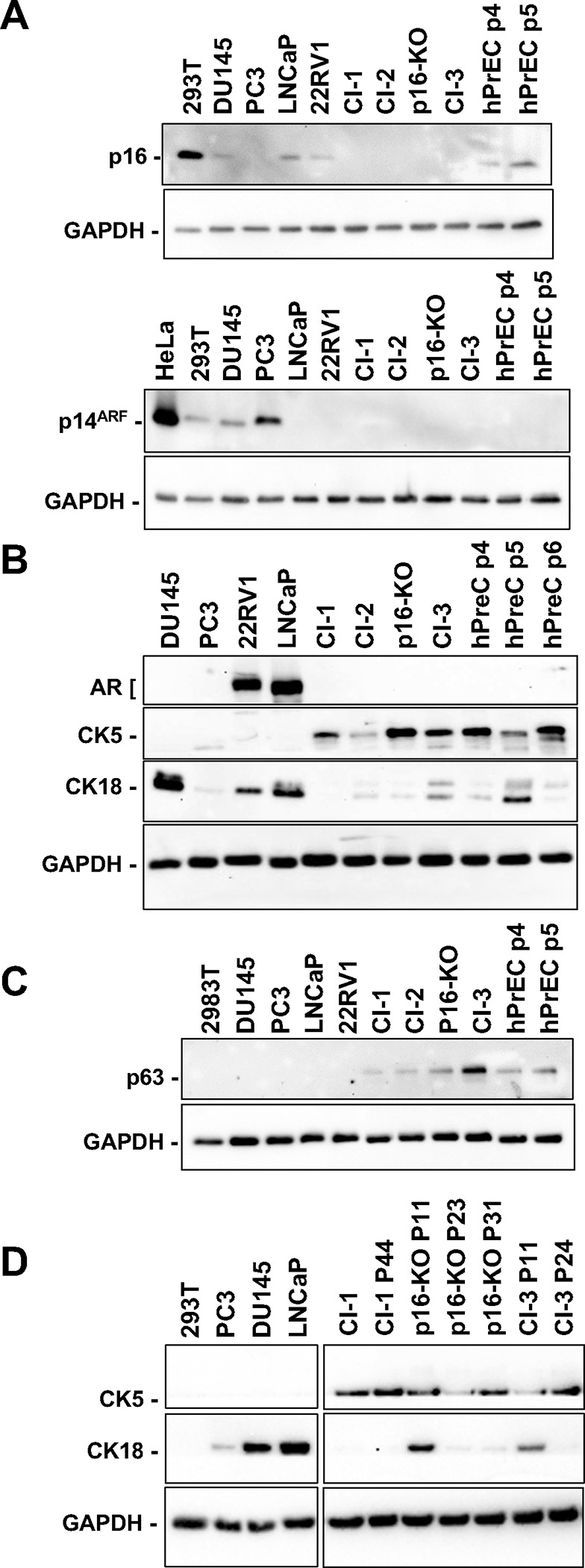
p16-KO and Cl-3 express basal markers and diminishing expression of the CK18 luminal marker with passage. Expression of p16^INK4A^, and p14^ARF^ **(A)** CK5 (basal marker), CK18 (luminal marker) and AR **(B)** and p63 (basal marker) **(C)** was determined by western blot analysis using whole cell lysates from the indicated cell lines and primary PrEC. **(D)** Expression of CK5 and CK18 through passages was determined for clones p16-KO and Cl-3. Relevant proteins are indicated on the left. Western blot images are cropped for clarity. Images corresponding to full-length membranes are shown in Suppl. Fig. 2.

**Figure 4.**
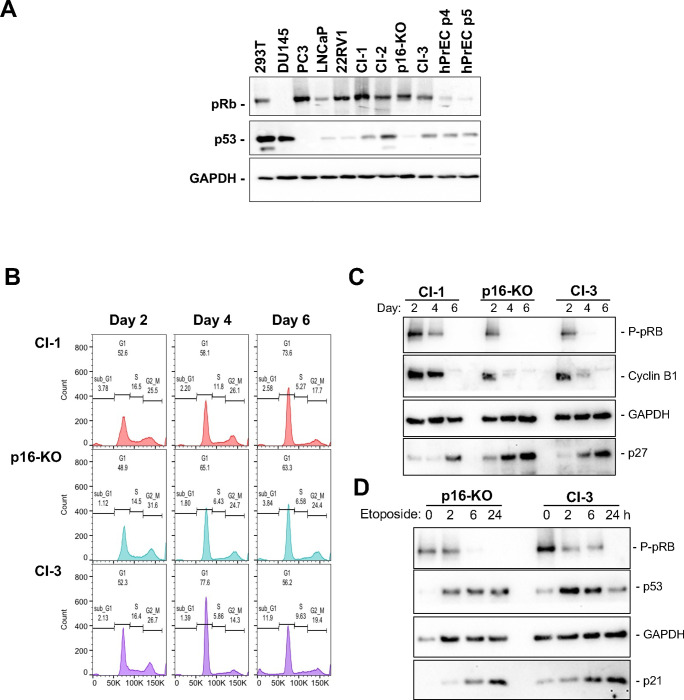
p16-KO and Cl-3 immortal cell lines exhibit properties of normal cells including contact inhibition and G1 checkpoint integrity. **(A)** All clones express pRB and p53 as determined by western blot analysis. **(B)** Growth to high cell density results in cell cycle exit. hPrEC Clone 1, and newly derived p16-KO and Cl-3, were grown to confluency. Cells were collected at the indicated times (in days) and cell cycle arrest was detected by measuring DNA content by PI/FACS analysis. **(C)** Expression of the p27 (high in G0, quiescence), cyclin B (G2/M marker) and phospho-pRB (P-pRB, mid G1 to M marker) were determined by western blot analysis. **(D)** p16-KO and Cl-3 cells have a normal response to DNA damage. Treatment with etoposide (60 μM), a cytotoxic agent, increased p53 and p21 expression as determined by western blot analysis. All experiments were performed at least in duplicate and a representative experiment is shown. Western blot images are cropped for clarity. Images corresponding to full-length membranes are shown in Suppl. Fig. 3.

We next sought to confirm that no protein products were expressed from the targeted *CDKN2A* exon 1α locus by western blot analysis. HEK-293T cells were used as a positive control for p16^INK4A^ and p14^ARF^ expression, and a panel of PCa cell lines, as well as our two previously derived hPrEC T-ΔN2A clones (Cl-1 and Cl-2), used as controls for the additional cell markers. We detected p16^INK4A^ expression in hPrEC at passage 5, but no expression of p16^INK4A^ was detected in the newly immortalized p16-KO or Cl-3 cell lines (Fig. 3A), suggesting that the CRISPR-mediated alterations of exon 1α or 2 efficiently prevented p16^INK4A^ expression. Expression levels of p14^ARF^ also reflected our CRISPR targeting, with a very faint signal detected in the p16-KO clone (in which p16^INK4A^ alone was knocked out), while no signal was detected in Cl-3 (in which both p16^INK4A^ and p14^ARF^ were targeted). Of note, p14^ARF^ expression was also not detected in hPrEC at passage 5, which likely reflects a lack of expression at early cell passages. These cells express p53 (Fig. 4A), which is likely triggered through a p14^ARF^-independent mechanism. In contrast, p14^ARF^ was readily detectable in PC3 and DU145 PCa cell lysates.

Next, we sought to assess the expression levels of cell-type specific markers in the two newly immortalized hPrEC lines. p16-KO and Cl-3 cells expressed the basal cell marker cytokeratin 5 (CK5) (Fig. 3B), which was also detected in hPrEC, Cl-1, and Cl-2. Consistent with these data, another known basal marker, p63, was detected in all the tested clones (Fig. 3C). As expected, neither basal marker was expressed in the DU145, PC3, 22RV1, or LNCaP cancer cell lines (Fig. 3B–C). Interestingly, albeit at low levels, we also detected expression of the luminal cell marker cytokeratin 18 (CK18) in all clones except Cl-1 (Fig. 3B). This could reflect a mixed population of prostate basal and luminal epithelial cells within the p16-KO, and Cl-3 cell lines, with the expression levels of cytokeratin 5 or 18 reflecting the proportions of each cell type within the clones. Supporting this possibility, we also observed a sharp decrease in CK18 expression with passage in p16-KO and Cl-3 (Fig. 3D).

### The newly-immortalized Immortalized cell lines retain the properties of normal parental PrECs.

To determine if the immortalized hPrEC lines retain key gate-keeping properties of normal cells, we sought to determine their response to signals that activate the pRB and p53 pathways. As expected p16-KO and Cl-3 cells expressed both pRB and p53 (Fig. 4A). Growth of normal cells to high cell density is known to result in a G0/G1 cell cycle arrest, which is mediated by dephosphorylation and activation of pRB and related proteins ^14,15^. To this end, p16-KO, Cl-3, and Cl-1 cells were seeded and allowed to grow until reaching confluency and beyond, while samples were longitudinally collected at the indicated times. Progressive accumulation of cells with a G0/G1 DNA content was observed, together with increased cell density. This was already observed at day 4, a time point that preceded cell confluence. Further, prominent G0/G1 arrest was observed by day 6 when cells were fully confluent (Fig. 4B). In agreement with these results, mitotic cyclin B1 expression and pRB phosphorylation were sharply downregulated, while the expression of the CDK2 inhibitor p27 was upregulated at 4–6 days (Fig. 4C). Consistently with these data we have observed that these cells are highly sensitive to cell-to-cell contact inhibition of proliferation and when seeded at low concentration, they form colonies that become quiescent even if these colonies do not fully cover the plate surface (data not shown). Thus, our results show that the cells are strongly arrested by cell contacts, which is consistent with these cells being derived from a non-transformed epithelial cell lineage.

We also interrogated whether the p53 pathway remained functional by treating cells with the topoisomerase II inhibitor, etoposide, in a time course of up to 24 h. As shown in Fig. 4D, p53 levels were strongly upregulated as early as 2 h following etoposide treatment, and this was followed by upregulation of its target effector p21, with maximal levels reached at 24 h. A concordant decrease in pRB phosphorylation was also detected.

Therefore, we concluded that both pRB and p53 functions remain intact when replicative and culture-induced senescence are eliminated through hTERT expression and p16 ablation.

### P4 PrEC and immortalized Clones 1, 2, 3, and p16-KO express surface epithelial and basal markers, but mostly lack expression of luminal markers.

Cl-3 and p16-KO express low levels of CK18 when compared to the luminal cell lines DU145 and LNCaP (Fig 2). However, CK18 levels in these immortalized PrEC clones are comparable to those observed in PC3 cells, which are known to be a luminal cancer cell line. We also observed that the levels of CK18 were increasingly limited with the passage in these clones (Fig. 2D). Given the apparent simultaneous expression of the two basal markers CK5 and p63 and the CK18 luminal marker, at least at lower passages, we could not conclusively distinguish their lineage of origin. Therefore, we used immunophenotyping with specific monoclonal antibodies, previously employed to recognize cell surface markers specific to luminal (anti-CD26) or basal (anti-CD271) prostate epithelial cells ^16^. Anti-CD326 was selected as a specific marker for epithelial cells. Also, to establish what distinct cell populations were present in the parental PrECs, we used tools from the *Human Protein Atlas* database that utilizes UMAP plots obtained from prostate tissue single-cell RNA-Seq (scRNA-Seq) data to distinguish cell populations ^17^ and expression patterns in prostate tissue cores ^18^. These tools show the expression of individual markers in prostate cells (Supplementary Fig. 2). The scRNA-Seq UMAPs define distinct populations of epithelial cells (basal: C2 and C3) and (glandular: C8 and C14), as well as subpopulations of endothelial, urothelial T cells and macrophages. As expected, basal (basal prostatic) and luminal (glandular) cells express the CD326 epithelial marker. Basal cells C3 expressed the CD271 marker (C2 to a lower extent), and the C8 population of granular epithelial cells expressed the luminal marker CD26. Of note, C14 glandular cells were mostly negative for the CD26 marker (Suppl. Fig. 4).

**Figure 5.**
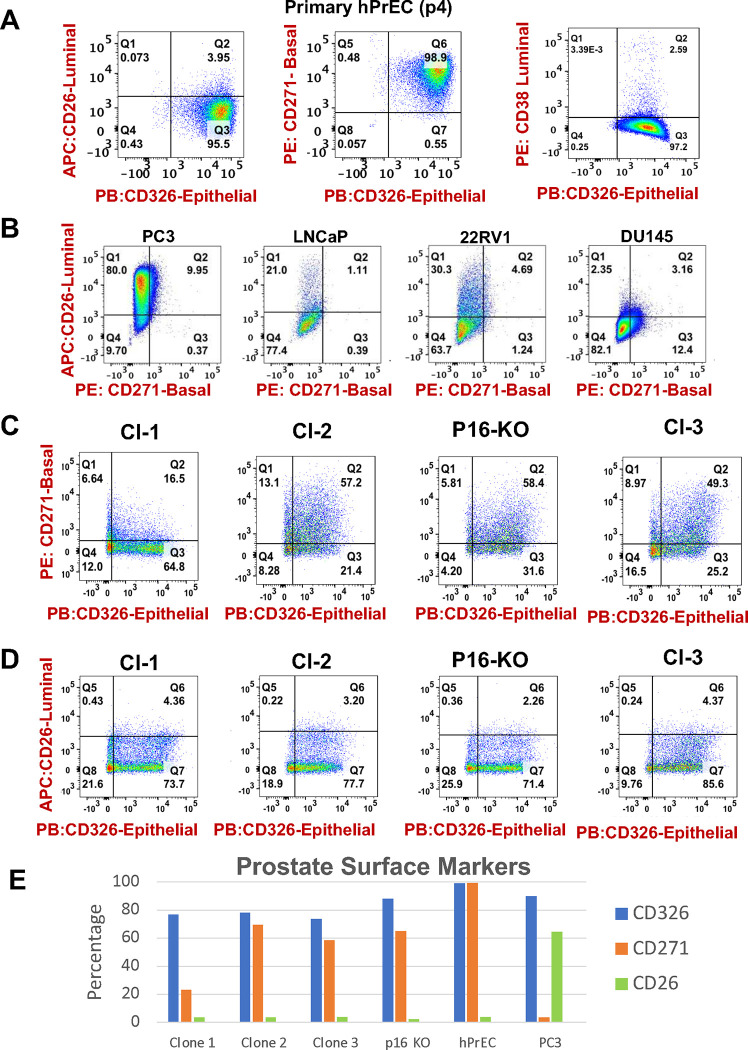
Expression of basal, luminal, and epithelial cell surface markers in hPrEC and derived clones. **(A)** Expression of CD326 (Epithelial) on the x-axis and CD26 (Luminal), CD271 (Basal) and CD38 (luminal) on the y-axis in primary hPrEC stained with the respective antibodies. **(B)** Expression of CD271 and CD26 on PC-3, LNCaP, 22RV1, and DU-145 PCa cell lines stained with the respective antibodies. **(C)** Expression of CD326 (on the x-axis) and CD271 (on the y-axis) in immortalized PrEC clones. **(D)** Expression of CD326 (on the x-axis) and CD26 (on the y-axis) in immortalized PrEC clones. **(E)** Bar graph depicting the percent of cells positive for the indicated markers quantitated from the data in A-D. All data were gated and analyzed in FlowJo.

Based on these data we predicted that immunophenotyping of early passage primary PrEC would reveal a large fraction of CD326 epithelial cells and that subfractions of these cells would be CD26 or CD271 positive. As expected, Fig. 5A (Suppl. Fig. 5A) shows that passage 4 PrEC stain >99.5% positive with anti-CD326 antibodies (x-axis). Surprisingly, most of these cells stained positive for the CD271 basal marker (y-axis, >99%), with very few cells expressing the luminal CD26 marker (<4%). In contrast and as expected, this antibody clearly stains luminal PC3 cells (Fig 5B, Suppl. Fig. 5B). We also determined the percent of CD26 positive cells across multiple PCa cell lines including DU-145, LNCaP and 22RV-1 all classified as predominantly luminal ^19^. Results showed that in contrast to PC-3 cells, a variable yet relatively small population of the analyzed PCa cells stained positive for CD26 (Fig. 5B). This strongly suggests that the CD26 antibody is not efficacious for identifying all the populations of luminal cells.

Using prostate scRNA-Seq data available from the *Human Protein Atlas,* we selected and tested another luminal maker, CD38, in which the RNA appears to be selective for C8 prostatic glandular cells, according to the UMAP plot (Suppl. Fig 4). Surprisingly, the CD38 antibody stained only ~2.59% of the parental PrEC at passage 4 (Fig. 5A and Suppl. Fig. 5A). While this could suggest that a small population of the PrEC express a luminal marker at passage 4, we should also restate that most of these cells are indeed positive for the basal marker CD271.

To assess the potential lineage origin, we next immunophenotyped the four PrEC clones, via flow cytometry. Upon staining with the respective antibodies, we observed that >74% of the cells are positive for the epithelial marker CD326, shifting the population to the Q2-Q3 quadrants (Fig. 5C, Suppl. Fig. 5C). A variable fraction of cells expresses CD271 in all clones. Cl-2 showed the highest percentage of CD271 positivity (~70%), followed by clone p16-KO and Cl-3 (64.8% and 58.5% of the cells, respectively). Cl-1 had the lowest percentage of basal cells at only 23%. In contrast, no expression of CD26 was apparent when compared to unstained controls for any of the clones (Fig. 5D, Suppl. Fig. 5D). A summary of surface markers is shown in Fig. 5E. Overall, this is consistent with the expression of CK5 and p63 detected by western blot analysis, with the majority of cells expressing basal cell marker CD271 (except in Cl-1), and no evidence of significantly mixed populations of basal and luminal cells in any of the clones as these are passed.

### RNA-Seq Analysis of PrEC immortalized Clones 1, 2, 3 and p16-KO strongly supports basal origin corresponding to population C2.

**Figure 6.**
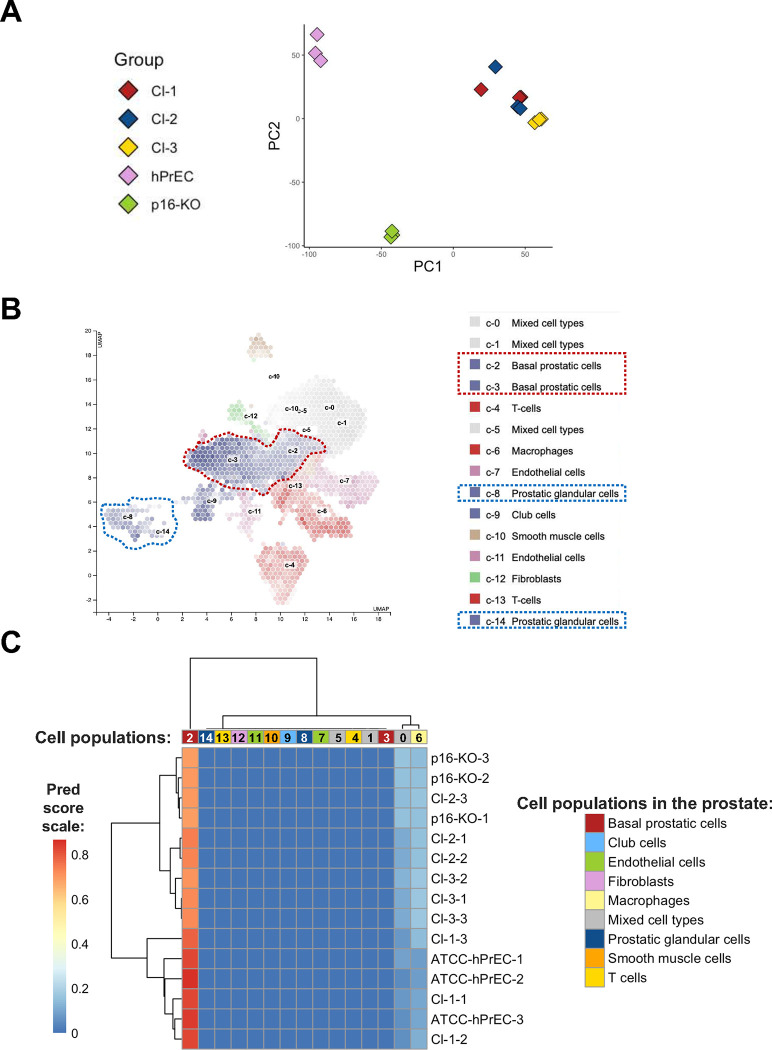
RNA-Seq Analysis showing gene expression and clustering of the immortalized cell lines and PrECs. **(A)** Principal Component Analysis **(**PCA) of the RNA-Seq data for all the clones and PrEC. (**B)** Prostate tissue UMAP plots obtained using scRNA-Seq by the Human Protein Atlas (with superimposed expression of Actin (ACTG1)) identify all distinct populations in the primary human prostate ^17^. **(C**) Gene expression heat map of predicted correlation scores (Preds) of each clone and hPrECs to the cell populations identified in the human prostate using scRNA-Seq. Prediction scores range from 0.0 to 1.0 (blue to dark red scale on the left). The distinct cell populations identified by the Human Atlas using single cell-RNA-Seq are labeled by color and a number (top of the heatmap and legend on the right). Clones and PrEC RNA-Seq samples were analyzed in triplicate. The prediction score in B indicate that the clones are most closely related to the basal prostatic population designated c2 (labeled red/orange in the first column of the heat map).

Given the limitations of measuring specific protein markers via western blot and immunophenotyping to define the prostate cell lineage that best represent these immortalized PrEC clones, we performed RNA-Seq analyses to compare their gene expression patterns to publicly available datasets of primary prostate cells ^17^. RNA was prepared for all clones and PrEC at passage 4. Principal Component Analysis (PCA) of all samples shows tight clustering of the three replicates for each sample (Fig. 6A). However, it grouped the parental hPrEC separately from the clones that were generated by targeting Exon 2 of the *CDKN2A* locus (Cl-1, Cl-2 and Cl-3) and the clone P16-KO (Fig. 6A). A heatmap shows the prediction scores for each prostate cell population identified by scRNA-Seq data obtained by the *Human Protein Atlas* (Fig. 6B) in each of the clones and PrEC (Preds) (Fig. 6C). The prediction scores show that PrEC and Cl-1 mapped closest with Human Atlas C2-basal prostatic cells (Pred >0.8) (Fig. 6C). Cl-2 Cl-3 and p16-KO cells were also highly correlated with the C2 population (Pred ~0.7). PrEC and all the clones had much lower correlations (Pred ~0.1) with a population of macrophages (C6) and a mixed cell type population (C0). Importantly, none of the clones, nor the PrECs, exhibited any correlation with the *Human Protein Atlas* C8 or C14 prostatic glandular clusters, from which only C8 express CD26 and CD38 (Suppl. Fig. 4). This potentially explains why we observed low expression of these luminal markers by immunophenotyping in the immortalized clones and the hPrECs at passage 4 (Fig. 5A). Thus, our RNA-Seq data agree with our immunophenotyping data and suggests that these luminal markers are not suitable for detecting all luminal cell populations.

**Figure 7.**
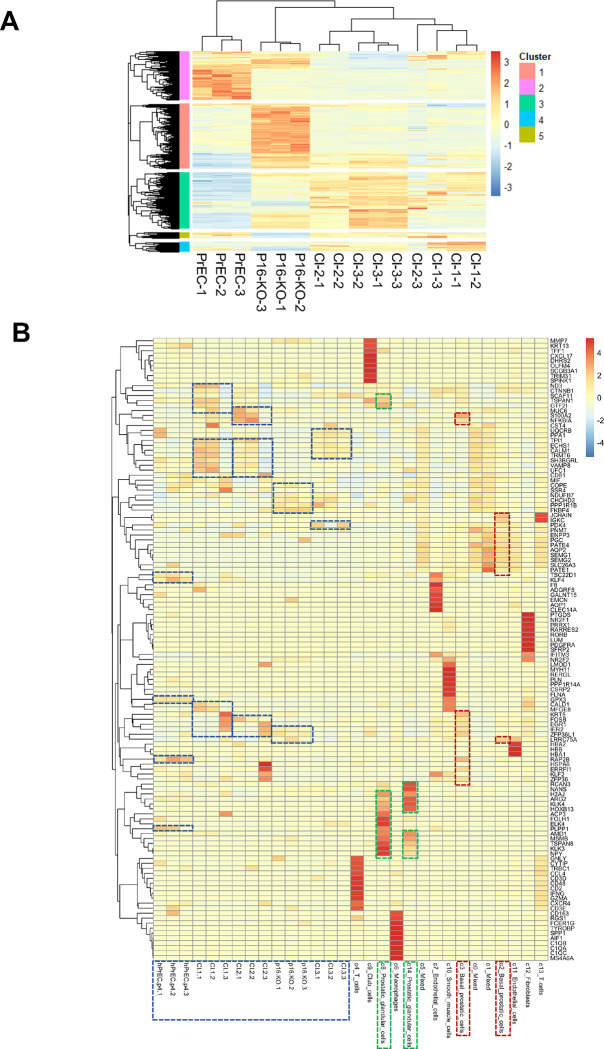
Heat maps of immortalized hPrEC clones in comparison to Sc-RNA-Seq data available on the *Human Protein Atlas* database. **(A)** heatmaps of differentiated expressed genes (DEGs), which identified 5 DEG clusters of genes with significant differences in expression (2-fold, p value <0.05) in at least one set of samples. **(B)** Heat map representing the top 5 genes in our samples in comparison to the RNA data defined by the *Human Protein Atlas* database. Note: this heat map lists 9 genes defined as N/A.

We then generated heatmaps of differentiated expressed genes (DEGs), which identified 5 DEG clusters of genes with significant differences in expression (2-fold, p-value <0.05) in at least one set of samples (Fig. 7A). Again, replicates clustered together, except for one Cl-2-3 replicate that was prepared through an independent batch. This heatmap also shows higher expression z-scores in subsets of genes for PrEC (clusters 2 and 3) and Clone p16-KO (clusters 1, 4, and 5) that cluster more distant from Clones 1, 2, and 3. Clones 1, 2, and 3 also have lower relative differences in gene expression among them (Fig. 7A). We used these data to query the expression of specific genes. As expected, and confirming the effectiveness of our approach, one of the top DEGs is *TERT,* which is shown as downregulated in PrEC relative to all the clones (Fig 2D). This is consistent with the forced expression of TERT in all the clones and is required for immortalization along with disruption of the *CDKN2A* locus. Unexpectedly, we did not detect statistically significant differences in the expression of full-length p16 or p14, which could reflect the low expression of these genes in PrEC or replicate variability that prevented reaching statistical significance.

Next, we generated heat maps with the top 10 DEGs across all four clones and PrEC compared to their expression in the cell populations identified by the *Human Protein Atlas* (Suppl. Fig. 4). The heatmap clearly shows clusters of genes specifically upregulated in the multiple cell populations from the primary prostate (C0-C14). Some of these genes are also expressed in the immortalized cell lines and in PrEC. However, we did not find a clear cluster of upregulated genes corresponding to a unique population of prostate cells defined by the *Human Protein Atlas*. This is not surprising, since prediction scores in Figure 6C show that the immortal cell lines highest correlation are with C2 basal prostatic cells. In C2 basal cells, their highest expressed genes (marked with a dashed red outline), are also highly expressed in mixed cell populations (C0, C1, C5). In Fig. 7B, it is also clear that there is a batch effect for our samples, as two replicates of our cell lines (Cl-1-3 and Cl-2-3) that were prepared separately from the other cell lines, show differential expression of certain genes including some that are high in C3 basal prostatic cells (marked with a dashed red outline). These expression differences in the cultured immortalized cells may reflect their differential growth status compared to primary cells in the prostate where only a small fraction of cells are proliferating. It could also reflect expression drifting due to culture conditions. This is already observable for the PrEC passaged in 2D culture, which do not exhibit high expression of genes that define their differentiated status in the prostate (marked with a dashed green or red outline).

### PrEC immortalized Clones 1, 2, 3, and p16-KO form round spheroids with a defined internal lumen in 3D Culture.

**Figure 8.**
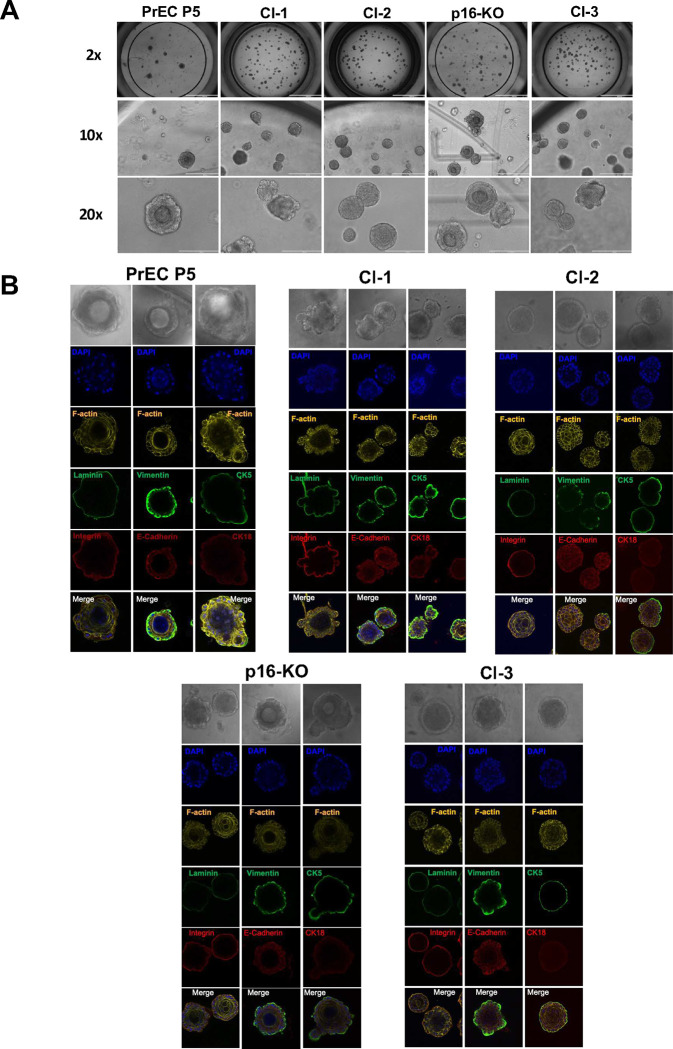
Comparative analyses of PrEC immortalized Clones 1, 2, 3 and p16-KO growing in 3D Cultures. **(A)** Representative images of each immortalized hPrEC clone and ATCC hPrEC p5 seeded in between two layers of Matrigel in iBidi μ-slide for 9–12 days. Organoid images taken using EVOS inverted microscope, at indicated fields. Note the formation of lumens in each clone. **(B)** Representative images of each PrEC immortalized clone and ATCC hPrEC p5 seeded between two layers of Matrigel and stained with laminin, vimetin (EMT marker), CK5 (Basal marker), Integrin B, E-cadherin (Epithelial Marker), CK18 (Luminal Marker). All organoids were also stained with phalloidin (F-actin) and DAPI. Images taken at 63x objective (Zoom factor .75) using Leica TCS SP8 Confocal Microscope.

We have previously shown that Cl-1 and Cl-2 efficiently form organoids when seeded between layers of Matrigel. These organoids express CK5 and very low levels of CK18 and are surrounded by basal lamina ^8^. Here we compared all clones to organoids formed by primary hPrEC at passage 5. Initial seeding of each cell line in μ-slides revealed that all the clones and the primary hPrEC can establish round organoids in Matrigel. To further characterize the size and shape of the organoids, daily monitoring and imaging of the same fields revealed the formation of distinct lumens over time in all clones (Fig. 8A, and data not shown). The resulting organoids were fixed after 9–12 days, and stained using specific antibodies, phalloidin to detect cytoskeletal actin filaments, and DAPI to note the nuclear DNA.

As expected, representative images show high CK5 expression at the boundary layer of spheroids facing the extracellular matrix (e.g., Matrigel). In contrast, very low levels of CK18 (luminal marker) were detected compared to background levels (Fig. 8B) as compared to secondary antibody alone (data not shown). As expected, all organoids generated a continuous “surrounding” basal lamina stained with anti-laminin antibodies that colocalizes with Integrin β1 positive areas, expressed solely by cells facing the ECM and suggesting a well-differentiated polarized organoid with a clear lumen. (Fig. 8B). Next, we used antibodies to Vimentin and E-Cadherin to asses any evidence of EMT, as well as differentiated epithelial cell-cell contacts, respectively. As expected, all the clones and PrEC showed an intense staining for Vimentin in cells localized at the basal layer. The distribution clearly differs from that of the integrin staining, which is restricted to the cell membrane (Fig. 8B). As expected, E-Cadherin stained at locations reminiscent of cell-cell contacts but was also detected at basal membrane cell layers as well as the lumen (Fig. 8B). Actin filaments were detected in the cytoplasm of all cells, mostly beneath the membrane. Overall clones and PrEC exhibited similar levels of expression and marker localization, but we noticed that the PrEC organoids appeared slightly less well-organized. However, this could reflect that the PrEC used in this experiment were at passage 5 (and heterogenous). At this passage, some cells may have already started to undergo senescence when grown in 2D. This could also explain why we obtained a fewer number of PrEC organoids when compared to all the other clones grown in 3D (Figure 8A).

## Discussion

Here we describe the derivation of immortalized normal prostate epithelial cells using a newly optimized methodology combining inactivation of *CDKN2A* exon 1α with co-expression of *hTERT*. This strategy spares inactivation of p14^ARF^, an unrelated product expressed from the *CDKN2A* locus. This is desirable because the resulting cells have an intact p53 pathway, and a pRB pathway that is only insensitive to senescence signals but fully active to integrate other growth inhibitory signals, such as those induced by increased cell density. Alterations of the *CDKN2A* locus are detected al low frequency in prostate tumors (~2%) ^20–23^. Thus, it is tempting to speculate that primary cells from tumors with alterations in the *CDKN2A* locus that already express hTERT may spontaneously establish in culture.

Characterization of the new clones of immortalized normal PrEC, along with those described by us previously ^8^, revealed that all of them express markers characteristic of basal prostatic cells (CK5, p63, and CD271). These cells also expressed low levels of the luminal marker CK18 in cells growing in both 2D and 3D culture conditions. Consistently, only a very small proportion of cells (~4%) expressed low levels of the commonly used luminal marker CD26. However, the analysis of the expression of this marker in PCa cell lines classified as luminal revealed major differences in the expression of CD26 and scRNA data from the *Human Protein Atlas* ^17^ identifies a population of non-basal granular prostatic cells that do not express CD26. In addition, bulk RNA-Seq analysis of all clones and PrEC revealed signatures that correlate best with one population of prostatic basal cells defined by the Human Atlas using scRNA-Seq (C2). Cl-1 and PrEC exhibited the highest prediction scores (~0.8) followed by the other clones, all with scores above 0.6. Of note the C2 population expresses low levels of the mRNA encoding CD271, which is detected in all our clones and the PrEC by FACS analysis (Fig. 6C). Consistent with a mixed population of prostatic epithelial cells, PrEC expressed CK5, p63, and CK18 at passage 4. However, very few cells expressed the CD26 luminal marker and only at comparatively low levels.

Multiple scenarios could explain these data: (i) cells derived from different individuals could vary in the expression of certain luminal markers (ii) CD26 and other luminal markers are downregulated as cells are grown in culture even when using optimal conditions, and (iii) luminal cells may senesce and/or die faster than basal cells under these optimal conditions. However, considering the high correlation between the gene expression signatures of PrEC with the C2 population of basal cells suggests that scenario 3 is more likely. Future studies using scRNA-Seq on populations of freshly isolated prostate cells vs. cultured cells at different cell culture passages should address all of these questions. This also supports the notion that the transduction of freshly isolated PrEC with *sgCDKN2A/Cas9* and *hTERT* lentiviruses is likely to allow the immortalization of a larger variety of cell populations.

In closing, this strategy could be used to build a collection of immortalized normal and PCa-derived cell lines from men with diverse genetic ancestry, which is lacking at present as only a few PCa cell lines are in existence today and are mostly derived from prostate tumors from men of Caucasian descent ^11,24,25^. In fact, a cell line named ACRJ-PC28 was recently derived from prostate cancer cells from an African-Caribbean patient using our previously reported methodology with simultaneous expression of hTERT and targeting *CDKN2A* exon 2 ^11^. Such cell lines could serve as cellular models to study in detail the stepwise transformation/tumorigenicity of human prostate epithelial cells by relevant oncogene/tumor suppressor genes, and genetic alteration cooperativity. These cells could also be used to study ECM invasion in 3D organoids as the result of specific genetic alterations. Moreover, the strategy could be applied to the rapid immortalization of cells in tumors and adjacent tumor tissue for studies of organoid formation and drug response, among others. Importantly, immortalized cell lines that retain the characteristics of the cells of origin are much easier to grow and cost-effective for long-term passage and further manipulation.

## Materials and Methods

### Cells and Cell Culture

**hPrEC** (Human Primary Prostate Epithelial Cells) cell were obtained from the American Type Culture Collection (ATCC cat # PCS-440-010) from a 16-year-old Caucasian male; Batch No (80619017). All tissues ATCC uses for isolation of primary cells are obtained under informed consent and conform to HIPAA regulations to protect the privacy of the donor’s Personally Identifiable Information. **Clone 1** (Cl-1, hPrEC-T-ΔN2A-1), **Clone 2** (Cl-2, hPrEC-T-ΔN2A-2), **Clone 3** (Cl-2, hPrEC-T-ΔN2A-3), **Clone p16-KO** (hPrEC-T-ΔN2A-1α), and primary hPrEC were cultured in Prostate Epithelial Cell Basal Medium (ATCC PCS-440-030) complete with Prostate Epithelial Cell Growth Kit components including 6 mM L-Glutamine, 0.4% Extract P, 1.0 mM Epinephrine, 0.5 ng/mL rhTGF-α, 100 ng/mL Hydrocortisone hemisuccinate, 5 mg/mL rh Insulin and 5 mg/mL Apo-transferrin (ATCC PCS-440-040). PC-3, DU14, LNCaP and 22RV1 cells were obtained and authenticated from Fox Chase Cancer (FCCC). PC-3 and DU14 cells were maintained in DMEM (Corning) containing 10% FBS and 100U/mL of Penicillin-Streptomycin. LNCaP and 22RV1 cells were maintained in RPMI 1640 medium supplemented with 10% FBS and 100U/ml of penicillin–streptomycin. All cells were grown at 37°C with 5% CO2.

### Immunophenotyping

Cell clones and ATCC hPrEC cells were collected with Acutase (GIBCO) and washed twice in flow buffer (5% FBS in PBS). Dry cell pellets were stained with the respective fluorophore-conjugated antibodies (see Table 1) in 100μl of flow buffer. Each sample was incubated in the dark for 20 minutes, then washed twice with 2 ml of flow buffer centrifuging at 200× g for 3 min. After final washes, the remaining flow buffer was removed, and each sample was fixed with 4% PFA incubating for 15 minutes in the dark. After fixation a final wash with 1 ml of flow buffer was done and all samples were resuspended in a final volume of 300 μl of flow buffer. Samples were protected from light and stored at 4°C until run on a BD LSR II or BD FACS ARIA II flow cytometer.

All experimental data were analyzed in FlowJo.

### Western Blot Analysis:

Cells were lysed in ice cold “E1A lysis” buffer (50 mM Tris-HCl (pH 7.4), 5 mM EDTA, 250 mM NaCl, 50 mM NaF, 0.1% Triton X-100) supplemented with (1 μg/mL aprotinin, 1 μg/mL leupeptin, 1 μg/mL Pepstatin A, 0.5 mM PMSF, 1mM β-glycerophosphate and 0.1 mM Sodium Orthovanadate) for one hour on ice. All subsequent steps were done at 4˚C or on ice. The protein concentration in whole cell lysates was determined using Bradford Assay (Protein Assay Dye, BioRad). 20 μg of protein per lane were resolved in 8–15% SDS–polyacrylamide gels and transferred to polyvinylidene fluoride (PVDF) membrane (Immobilon-FL, Millipore) in 1X CAPs buffer (0.1M CAPS, 10% Methanol, pH 11.00) and blocked in 5% non-fat dry milk or 5% BSA in 1x TBS-T for 1 h at room temperature. Membranes transferred from SDS-PAGE gels were cut horizontally in three pieces (top, middle and bottom) using protein ladders on the flanking lanes as guide. Membranes with blotted proteins in a molecular range were incubated with specific antibodies to a target protein in that molecular mass range (see Table 2) in 3% non-fat dry milk overnight at 4°C and washed with TBS-T (137 mM NaCl, 20 mM Tris, 0.5% Tween20, pH 7.6) then incubated with secondary antibody for 1 h, washed with TBS-T. Western Pico Plus (34580) ECL reagent was added to the membranes and visualized using a chemiluminescent imager.

### PCR

To verify *CDKN2A* ablation or mutation from immortalized clones, PCR was performed using primers targeting Exon 1α and Exon 1β or Exon 2 of the CDKN2A locus with annealing temperature at 55°C: ***CDKN2A* Exon 2 FWD**: CTG TGC TGG AAA ATG AAT GC. ***CDKN2A* Exon 2 REV**: CTG GAA GCA AAT GTA GGG G. ***CDKN2A* Exon 1α FWD:** TCC AGA GGA TTT GAG GGA CA. ***CDKN2A* Exon 1α REV:** GCT CCT CAT TCC TCT TCC TTG. ***CDKN2A* Exon 1β FWD:** GGT CCC AGT CTG CAG TTA AGA. ***CDKN2A* Exon 1β REV:** CTG ACT TCT GAG GTG GGT TTA G. Globin primers were used as control. **Globin FWD**: CAA CTT CAT CCA CGT TCA CC. **Globin REV**: GAA GAG CCA AGG ACA GGT A.

#### RNA Libraries Preparation

Cells were collected, and RNA was isolated by Qiagen RNeasy kit (Cat. No. 74104). RNA libraries were prepared in triplicate using the NEBNext^®^ Ultra^™^ Directional RNA Library Prep Kit from Illumina (Cat #E4720L) and sequence at the Fox chase Cancer Center using a Nextseq2000 sequencer. 100–1000ng total RNAs from each sample were used to make libraries. mRNAs were enriched twice via poly-T based RNA purification beads and subjected to fragmentation at 94 °C for 15 min via divalent cation method. The 1^st^ strand cDNA was synthesized by reverse transcriptase and random primers at 42 °C for 15 mins, followed by 2^nd^ strand synthesis at 16°C for 1hr. During second strand synthesis, the dUTP was used to replace dTTP, thereby the second strand was quenched during amplification. An ‘A’ nucleotide is added to the 3’ ends of the blunt fragments at 37°C for 30 min. Adapters with illumine P5, P7 sequences as well as barcodes were ligated to the cDNA fragment at 30°C for 10 min. After SPRIselectbeads (Beckman Coulter, Cat# B23318) purification, a 15-cycle PCR reaction was used to enrich the fragments. PCR was set at 98°C for 10 sec, 60°C for 30 sec and extended at 72°C for 30 sec. Libraries were again purified using SPRIselectbeads, validated on a Agilent 2100 bioanalyzer (serial # DE34903146) using Agilent high sensitive DNA kit (Cat# 5067-4626), and quantified with Qubit 3.0 fluorometer (ThermoFisher Scientific, Cat#Q33216) using Qubit 1x dsDNA HS assay kit (Cat#Q33230). Paired end reads at 65 bp were generated by using Nextseq 2000 high output reagent kit v2.5 (Illumina, Cat# 20024907). Fastq files were obtained at Illumina base space (https://basespace.illumina.com)

#### RNA-Seq Analysis:

To generate PCA Plots, FASTQ files from the sequenced libraries were aligned to the genome with STAR aligner and gene expression for each gene was counted. DESeq2 was used to normalize the expression across all samples. The correlation Value heat map was generated using R package Seurat. Seurat was used to calculate the confidence in which differentially expressed genes (DEGs) from the four clones and PrEC were correlated with the Human Protein Atlas populations defined by scRNA-Seq. This resulted in a matrix showing the correlation scores, which was visualized as heatmap. The Heat Map of DEGs were created in R and DEGs shown are significant across all samples. Significant DEGs were featured in the heatmap and arranged via hierarchical clustering, grouping them according to their expression across samples. The top 10 DEGs in the four clones and PrEC were compared to the top 10 DEGs in the prostate populations defined by scRNA by the Human Protein Atlas. Hierarchical clustering generated a heat map for the top 10 significantly expressed genes in both data sets. Top 10 DEGs in the four clones, PrEC and the prostate populations defined by scRNA by the Human Protein Atlas were selected based on each gene’s fold change in each sample compared to the average expression across the dataset. DEG test across the samples also considered whether the data came from bulk or scRNA-seq, as a variable regressed out in a linear model to determine DEG significance. Finally, for visualization, the heatmap used a matrix with counts adjusted based on the sequencing type, corrected via combat in R’s sva library.

### Organoid Culture

For 3D organoid culture, procedures were adapted from ^26^. For hPrEC organoids, Growth Factor Reduced Matrigel Matrix (Corning, REF 356231) and for PC3 cells Basement Matrigel (BD, 354234) were thawed overnight at 4°C. 10 μl of 40% Matrigel was added to the bottom of iBidi μ-slide (ibidi#81501) and incubated at 37°C for at least 30 min. Generated clones and ATCC hPrEC cells were detached by Accutase cell detachment solution (Cat-Corning-25-058-Cl) and 200 cells were counted and seeded on top of the 40% layer of Matrigel. Cells were left to attach for at least 2 hours at 37°C. The medium was then slowly removed and 20 μl of 20% Matrigel was added as another layer to the cells. After solidifying, 25–30 μl of medium was added and replaced every other day.

### Immunofluorescence Staining of 3D culture

After 9–10 days, organoids were washed with cold PBS before fixation. 30 μl of fixation/permeabilization solution was added, consisting of 2% paraformaldehyde, 0.6% TritonX-100, 5 mM EGTA, 1 mM MgCl_2_ in PBS. After fixing at room temperature for 20 min, the fixation/permeabilization solution was removed. Organoids were washed with PBS twice, then blocked in 20% horse serum in PBS-T (PBS with 0.05% Tween 20) at room temperature for an hour. 20% horse serum was removed, and samples were incubated with primary antibodies diluted in PBS-T for 60 min at room temperature or at 4°C overnight. Wells were washed with PBS twice, followed by fluorescence-conjugated secondary antibodies mixed with phalloidin diluted in 1% BSA for an hour at room temperature. After rinsing twice with PBS, DAPI was applied to samples for 30 min, then washed with PBS-T twice and 25 μl of fresh PBS-T was added to each well. Samples were protected from light until confocal analysis was performed using Leica TCS SP8 confocal microscope.

## Figures and Tables

**TABLE 1:** 

Antibody	Company	Catalog#	Application
			
**APC Mouse Anti-Human CD26** **Clone M-A261**	BD BioScience	563670	FC
**APC Cyanine 7 Anti-human CD38** **Clone HIT2**	BioLegend	303533	FC
**BV-421 Anti Human CD326 Ep-CAM** **Clone 9C4**	BioLegend	324219	FC
**Pacific Blue Anti Human CD326 EP-CAM** **Clone 9C4**	Biolegend	324217	FC
**PE Anti-human CD271 NGFR** **Clone ME20.4**	BioLegend	345105	FC

**TABLE 2:** 

**Primary Antibody**	**Company**	**Catalog#**	**Application**
			
**GAPDH**	Santa Cruz	sc-47724	WB
**Keratin 5**	BioLegend	905503	WB, IF
**Keratin 18**	BioLegend	628401	WB, IF
**p14ARF**	CST	74560S	WB, IHC, IF
**p16**	Santa Cruz	sc-468	WB
**p53**	Santa Cruz	Sc-126	WB
**p63**	Santa Cruz	sc-25268	WB, IF
**P-pRb, phospho-Rb (Ser807/Ser811)**	CST	8516	WB, IF
			
**Secondary Antibody**	**Company**	**Catalog#**	**Application**
			
**ECL Rabbit IgG, HRP-linked whole Ab (from donkey)**	GE Healthcare	NA934V	WB
**ECL Mouse IgG, HRP-linked whole Ab (from sheep)**	GE Healthcare	NA931V	WB

**TABLE 3:** 

**Primary Antibody**	**Company**	**Catalog#**	**Application**
			
Integrin b1	Santa Cruz	sc-59827	IF
Laminin b1	Santa Cruz	sc-33709	IF
Vimentin	abcam	ab92547	IF
E-Cadherin	CST	14472S	WB, IF, IHC
Keratin 5	BioLegend	905503	WB, IF
Keratin 18	BioLegend	628401	WB, IF
Phalloidin-iFluor 555 Conjugate	abcam	ab176756	IF
			
**Secondary Antibody**	**Company**	**Catalog#**	**Application**
			
Goat anti-Rat IgG (H+L) Cross-Adsorbed Secondary Antibody, Alexa Fluor 488	Invitrogen	A11006	IF
Goat anti-Rabbit IgG (H+L) Highly Cross-Adsorbed Secondary Antibody, Alexa Fluor 488	Invitrogen	A11034	IF
Goat anti-Mouse IgG (H+L) Cross-Adsorbed Secondary Antibody, Alexa Fluor 647	Invitrogen	A21235	IF

## Data Availability

The RNA-Seq data described in this manuscript is available upon request to the corresponding author (XG).
